# American Dental Society of Europe

**Published:** 1894-10

**Authors:** Wm. S. Davenport

**Affiliations:** Secretary


### AMERICAN DENTAL SOCIETY OF EUROPE.

The nineteenth meeting of this Society was held at Geneva, August 6 to 8. Dr. Royce, of Tunbridge Wells, England, reported experiments made upon puppies of two weeks old to test the effect of the direct action of mercury upon the formation of enamel. He advanced the theory that the defects ascribed by Hutchinson to hereditary syphilis may rather be attributed to hereditary mercurialization. His experiments in this direction are not yet complete, but will be awaited with interest.

Papers were also presented by Professor W. D. Miller, of Berlin, Dr. Luce, of Stuttgart, and others, and Professors Eternod and Vulliet, of Geneva University, gave interesting demonstrations, the former on the "Law of Statics in Hard and Soft Dental Tissues," the latter on "Dermoid Growths," which were followed with great interest by all the members.

The following officers were chosen for the year 1894–95: President, Dr. Chas. W. Jenkins, Zürich; Vice-President, Dr. Wm. Mitchell, London; Treasurer, Dr. Chas. J. Monk, Wiesbaden; Secretary, Dr. Wm. S. Davenport, Paris.

The next meeting will be held at Boulogne, France.

WM. S. DAVENPORT,
*Secretary.*

